# Obituary

**Published:** 1893-01

**Authors:** 


### Obituary.

Dr. A. Reeves Jackson died at his home in Chicago, November 12, 1892. He was Professor of Gynecology in the Chicago College of Physicians and Surgeons, and was one of the best known gynecologists in the West. Dr. Jackson's personal magnetism and genial disposition won him a large group of friends, both within and without the ranks of the profession, and it will not be easy to fill the social and professional place that is so sadly vacated.

---

The positions of House Physician and assistant, at the Buffalo City Hospital, will be vacant March 1, 1893. All graduates in medicine desiring to compete for these positions may obtain information by addressing Dr. John Pryor, President, or Dr. Wm. C. Krauss, Secretary of the staff.

